# Genome‐wide genotyping of a novel Mexican Chile Pepper collection illuminates the history of landrace differentiation after *Capsicum annuum* L. domestication

**DOI:** 10.1111/eva.12651

**Published:** 2018-07-11

**Authors:** Nathan Taitano, Vivian Bernau, Lev Jardón‐Barbolla, Brian Leckie, Michael Mazourek, Kristin Mercer, Leah McHale, Andrew Michel, David Baumler, Michael Kantar, Esther van der Knaap

**Affiliations:** ^1^ Institute for Plant Breeding, Genetics & Genomics University of Georgia Athens Georgia; ^2^ Department of Horticulture and Crop Science Ohio State University Wooster Ohio; ^3^ Department of Horticulture and Crop Science Ohio State University Columbus Ohio; ^4^ Center of Interdisciplinary Research in Sciences and Humanities Universidad Nacional Autónoma de México Mexico City México; ^5^ Section of Plant Breeding and Genetics Cornell University Ithaca New York; ^6^ Department of Entomology Ohio State University Wooster Ohio; ^7^ Department of Food Science and Nutrition University of Minnesota Minneapolis Minnesota; ^8^ Department of Tropical Plant and Soil Sciences University of Hawai'i Honolulu Hawaii; ^9^ Department of Horticulture University of Georgia Athens Georgia; ^10^ Present address: School of Agriculture Tennessee Technological University Cookeville Tennessee

**Keywords:** domestication, fruit morphology, GBS, genetic diversity, landrace, pepper

## Abstract

Studies of genetic diversity among phenotypically distinct crop landraces improve our understanding of fruit evolution and genome structure under domestication. Chile peppers (*Capsicum* spp. L.) are economically valuable and culturally important species, and extensive phenotypic variation among landraces exists in southern Mexico, a center of *C. annuum* diversity. We collected 103 chile pepper seed accessions from 22 named landraces across 27 locations in southern Mexico. We genotyped these accessions with genotyping by sequencing (GBS), yielding 32,623 filtered single‐nucleotide polymorphisms. Afterward, we genotyped 32 additional *C. annuum* accessions from a global collection for comparison to the Mexican collection. Within the Mexican collection, genetic assignment analyses showed clear genetic differentiation between landraces and clarified the unique nature of the Tusta landrace. Further clustering analyses indicated that the largest fresh‐use Chile de Agua and dry‐use Costeño landraces were part of separate clades, indicating that these two landraces likely represent distinct populations. The global accessions showed considerable admixture and limited clustering, which may be due to the collapse of use‐type divisions outside of Central America. The separation of the Mexican landraces in part by fruit morphology related to use highlights the relevance of this use‐type morphological diversity for plant breeders and the utility of fruit development variation for evolutionary biologists.

## 1 INTRODUCTION

Evolutionary biologists have been interested in domesticated plants to study natural selection for more than a century (Darwin, 1868). Just like any other plant system, crop populations can be subject to the structure‐reducing effects of gene flow and the structure‐enhancing effects of genetic drift, selection, or assortative mating (Loveless & Hamrick, 1984). However, cultivated populations have unique characteristics as breeding may accelerate local adaptation. In this case, human management can create reproductive isolation, by the removal of phenotypically distinct individuals (rogueing) from homogenous cultivated plant stands, or by purposefully isolating distinct types into separate stands to prevent outcrossing. Landraces grown near a crop center of origin present ideal populations in which to study crop diversification and genetic structure, as well as the presence of long‐standing populations derived soon after domestication (Zeven, 1998).

Levels of genetic structure in domesticated populations are largely determined by the diversifying effect of population isolation (e.g., of specialized landraces) balanced against the homogenizing effect of gene flow and the planting of homogenous elite cultivars. Smallholder farmers may also strive to retain traditional varieties on small plots in their farms while participating in germplasm exchange that includes productive modern cultivars (e.g., in the Andes; Brush, Taylor, & Bellon, 1992). As a result, landraces (e.g., Andean potatoes, *Solanum spp*.) near their center of origin can retain a high level of diversity on individual farms, even as regional diversity diminishes (Zimmerer & Douches, 1991).

Another factor that affects the level of genetic diversity found in a crop population is its level of domestication. The degree of domestication is difficult to measure precisely. For the purposes of this study, which examines chile peppers (*Capsicum spp*. L.) we have grouped the domestication level of different seed accessions into four categories of cultivation. From least domesticated to most domesticated, these are accessions produced in the forest, backyard, *milpa*, and plantation environments. Forest‐grown populations may be collected by the community, but they are rarely intentionally planted and exist outside of an intentionally cultivated environment; thus, they likely represent the least domesticated types. Backyard populations, which cover let‐stand populations and those casually cultivated in backyard gardens, represent a level of human cultivation and domestication that is relatively unrestrictive. Even though there may be a moderate level of selection, these populations are likely not subjected to the rigorous scrutiny imposed on peppers destined for market. In Mesoamerica, a *milpa* is a cultivated maize field that often incorporates intercropping of other species, such as beans, squash, and chile peppers. Some *milpas* include agroforestry components and mirror natural, postdisturbance, forest succession (Nigh & Diemont, 2013). Although actual *milpa* practices may vary among farmers, the higher species diversity and forest proximity inherent to *milpa* environments attracts generalist pollinators that mediate pepper pollination, outcrossing, and fruit set (Landaverde‐González et al., 2017; Raw, 2000). We define plantations as agricultural systems where a single crop is planted in rows, usually of a single variety that is saved year to year by the farmer. This is the most restrictive domestication environment.

The chile pepper, especially *Capsicum annuum,* is a widely cultivated species with many phenotypically diverse landraces (Bosland & Votava, 2012), and a well‐suited study system for exploring the genetic structure of landraces during their diversification. To date, limitations in data resolution (of markers or populations) have prevented studies from elucidating the genetics of diversification in *C. annuum*. Early studies were limited by genomic resolution as only a dozen or fewer markers were employed for the analyses (Hernández‐Verdugo et al., 2001; González‐Jara, Moreno‐Letelier, Fraile, Piñero, & García‐Arenal, 2011), as it was difficult to generate markers for the large pepper genome (3.48 Gb; Qin et al., 2014). More recent studies sampled many, mainly elite populations with few representatives of each population (Hill et al., 2013; Hulse‐Kemp et al., 2016; Naegele, Mitchell, & Hausbeck, 2016). While the latter studies demonstrate the genetic diversity that is relevant to elite germplasm, the degree of genetic diversification among long‐standing *C. annuum* landraces is unknown. Thus, despite evidence that pepper has been cultivated for thousands of years (Perry et al., 2007; Perry & Flannery, 2007), more comprehensive sampling and genotyping, with improved genomic coverage, may better elucidate the processes of diversification under domestication.

This study sampled chile pepper populations from the Southern Mexican states of Oaxaca and Yucatan. *C. annuum* is of cultural importance in Mexico, especially in Oaxaca, where it exhibits dramatic genetic and phenotypic diversity. Early chile pepper depictions show that cultivated *C. annuum* fruits were much larger than their wild counterparts and had an array of uses spanning many hundreds of years (Codex Mendoza, 1542). This diversity of uses capitalizes on a diversity of chile pepper “use‐types,” that is, assemblages of potentially related plants bearing fruits with distinct morphological characteristics well suited for their particular use, which are also found in archeological remains (Perry & Flannery, 2007). Oaxaca spans a range of climates, owing to its sharp elevation gain inland from the coast, as well as precipitation differences along the coastline (Fick & Hijmans, 2017). Moreover, Oaxaca has been called the most ethnically diverse state in Mexico, home to more than 16 languages (Romero, 2000). Thus, high climate and cultural diversity make Southern Mexico a center of great diversity for chile peppers and a likely center of domestication for chile peppers (Aguilar‐Meléndez, Morrell, Roose, & Kim, 2009; Kraft et al., 2014). In summary, chile peppers from Southern Mexico are an ideal target to analyze long‐standing landrace populations for patterns of genetic diversity created since domestication. In order to extend our study of genetic diversity to peppers grown outside its center of diversity in southern Mexico, we included chile peppers collected from around the world (Kantar et al., 2016).

Our objectives were to characterize the genetic diversity among the Oaxacan landraces and compare them to accessions from around the world. Understanding of the genetic diversity in these chile peppers can lead to populations that may contain potentially useful alleles that were missed when selecting germplasm to develop into modern cultivars.

## 2 MATERIAL AND METHODS

### 2.1 Study system

The genus *Capsicum* is a member of the agriculturally important Solanaceae family, which also includes potato, tomato, eggplant, and tomatillo. After branching off from the tomato and potato lineage *c*. 36 million years ago (Qin et al., 2014), the *Capsicum* lineage itself diverged into over a dozen species (McLeod, Guttman, & Eshbaugh, 1982). Of these, five species were domesticated in Central and South America: *C. baccatum* L., *C. pubescens* Ruiz & Pav.*, C. frutescens* L.*, C. chinense* Jacq.*,* and *C. annuum*. The latter three are relatively interfertile with each other and form the “*Capsicum annuum* complex” (Pickersgill, 1988). *C. annuum* makes up the majority of varieties now cultivated worldwide (Bosland & Votava, 2012). All these varieties are descended from *C. annuum* originally domesticated in present‐day Mexico (Kraft et al., 2014), with remains in the Tehuacán valley dated to *c*. 6,000 years ago, about 1,000 years after general crop cultivation began in this area (Brown *et al*., 2013; Smith, 1997).

### 2.2 Plant materials

**Figure 1:**
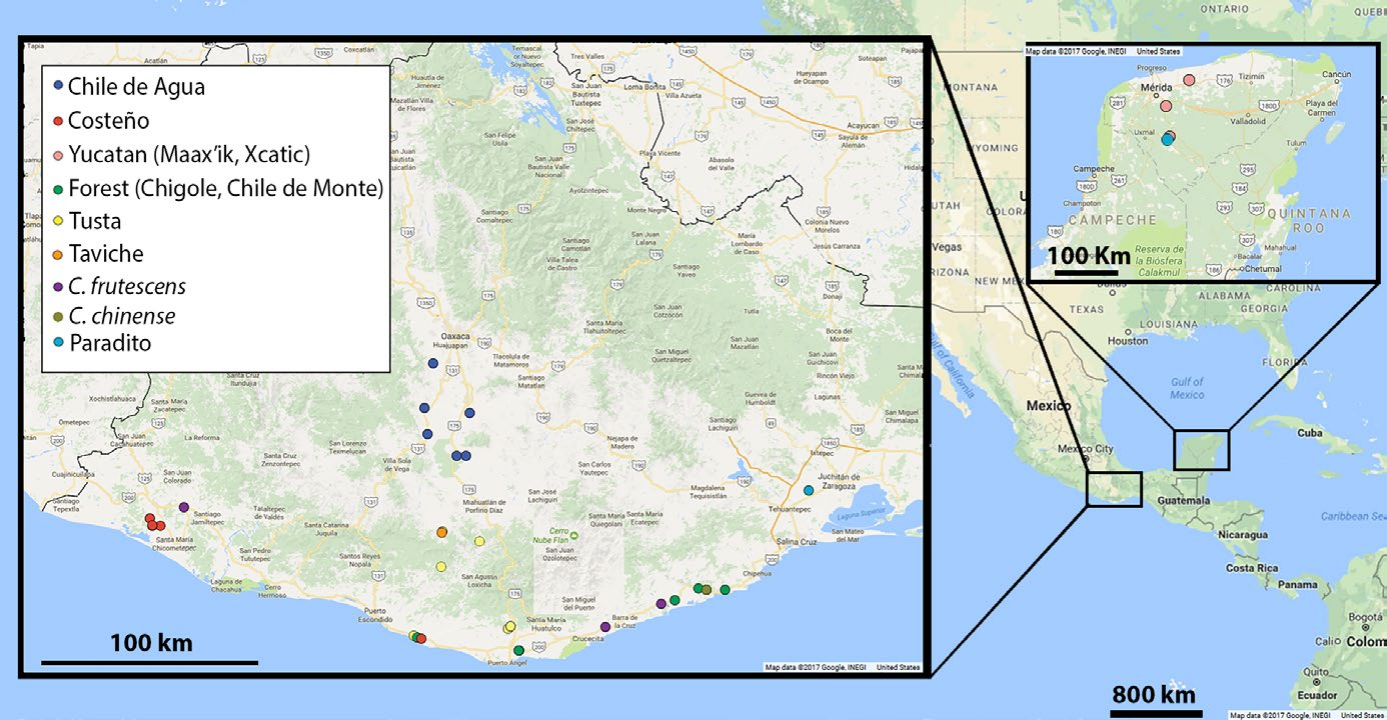
Map of chile pepper collection sites in the Mexican states of Oaxaca and Yucatan. The colored circles highlight the most common type grown at that site. Larger image shows Oaxaca and inset map at top right shows Yucatán

Pepper accessions were collected in 2013 from two overlapping transects in Oaxaca. These collection sites allowed us to sample the major sources of variation among landraces that are present in the region (Figure 1). The first transect encompassed 13 sampling locations and ran north–south along an elevation and temperature gradient, from the central valley's near Oaxaca City, *c*. 1,500 m above sea level (masl) to the southern tip of the Pacific coastline in Pochutla (<600 masl). There, it borders the coast and the second transect. The second transect ran east–west along the Pacific coast, which spanned a precipitation gradient and included twelve sites. Both transects spanned ethnic and language groups. The peppers collected from three sites in the Yucatán were from the villages Maní, Acanceh, and Cansahcab. In total, we collected seed from 27 different locations in Mexico. Together, these peppers will be referred to throughout this study as the “Mexican collection” (Supporting Information Table S1). From the Mexican collection, 103 accessions produced viable seed from which two seedlings were grown where possible, ultimately yielding 190 plants which were genotyped for this study. Those plants were grown in a Columbus, OH greenhouse in 2014 in a completely randomized design.

The pepper collection from around the world was obtained from heirloom seed producers in North America (see Kantar et al., 2016). They originated from multiple geographies and contained *C. annuum* landraces and cultivars. This collection of chile peppers from around the world is henceforth referred to as the “global collection,” and only accessions with sufficient read depth were used for this study. After germination and growth indoors for 9–14 weeks, plants from this collection were transplanted into five‐gallon containers and grown outdoors in a completely random design with two replications in Madison, WI during the summer of 2013. Young leaves from each plant were harvested and frozen at −20°C for subsequent DNA extraction. Images of fruits were collected for each of the accessions and the major named types.

### 2.3 DNA Extraction

For the global accessions, gDNA from each was extracted by grinding 100 mg of frozen leaf tissue using dry ice, stainless steel beads, and a tissue homogenizer (Bullet Blender), then isolating DNA using the Omega Biotec E.Z.N.A. ^®^ Plant DNA Kit. Extracted DNA was stored at −20°C and sent to BGI Americas for GBS library construction and sequencing.

For the Mexican collection, DNA was collected from young leaves of adult chile pepper plants. Approximately 50 mg leaf sample from each plant was collected in deep‐well tubes in two 96‐well plates. The samples were stored on ice until collection was complete, then flash‐frozen in liquid nitrogen and lyophilized prior to DNA extraction. After lyophilizing, the samples were ground to a fine powder by adding metal beads and mechanically shaking in a Geno/Grinder 2000^®^ (SPEX, Metuchen, NJ, USA). DNA extraction was performed using QIAGEN's DNEasy 96 Plant Kit^®^ (Valencia, CA, USA), following the manufacturer's recommendation. DNAs were eluted into 100 μl TE pH 8.

### 2.4 Genotyping‐by‐sequencing library construction

Genotyping‐by‐sequencing (GBS) libraries were created following the established method (Elshire et al., 2011). To briefly summarize, genomic DNA was digested with the ApeKI methylation‐sensitive 5 base‐pair (bp) recognition site restriction enzyme. The resulting fragments were ligated to Illumina sequencing adapters and to adapters with sequence “barcodes” unique to each individual sample, enabling the recovery of source plant identity for each sequenced DNA fragment after multiplexing. For the Mexican collection, GBS libraries were constructed for each genotype and 48 libraries were pooled, size selected to an average size of 350 bp in length and sequenced. Two pools were sequenced on the NextSeq platform from which we obtained 725 million 75‐bp single‐end sequence reads and an average per‐individual coverage of 3.59X. Two additional pools were sequenced on the HiSeq 2500 platform for a total of 397 million 100‐bp single‐end reads, and an average per‐individual coverage of 1.96X. This resulted in an average of 344 million reads obtained per lane (2X expected coverage per reduced genome), with an average PHRED‐scaled quality score of 35 for the “Mexican collection.” On the other hand, the “global accessions” sequenced on the Illumina Hiseq 4000 at a 100 × 2 bp paired‐end read length yielded 19.5 million reads total for those individuals retained after quality control, for an average per‐individual coverage of 0.219X.

### 2.5 SNP calling

After a quality‐control step with FastQC (Andrews, 2010) and removal of poor quality reads, the TASSEL GBS Pipeline 5.2.3 (Glaubitz et al., 2014) was used to call single‐nucleotide polymorphisms (SNPs) from Illumina sequence data. The *C. annuum* cv. CM334 reference genome was used for read alignment with Bowtie2 (Langmead & Salzberg, 2012); a minor allele count of three reads per minor SNP allele was required to call a SNP (Supporting Information Appendix S1).

This SNP‐calling process returned a genotype table, which was then filtered prior to analysis using VCFTools (Danecek et al., 2011) to a list of biallelic SNPs that were excluded on the basis of the proportion of missing data, minor allele frequency, and the proportion of heterozygosity at each locus. Filtering thresholds for these metrics were set at ≤10%, ≥5%, and ≤10%, respectively, for the Mexican collection, and ≤20%, ≥1%, and ≤5% for the combined Mexican and global collection dataset. Thresholds were determined by plotting the metric for each SNP on the *y*‐axis, with the SNPs ordered by their value for that metric along the *x*‐axis, and visually identifying an inflection point in the resulting curve, which signified a sudden divergence in value for that metric from the baseline SNPs. Furthermore, to obtain markers for analyzing the combined Mexican and global dataset, SNPs were selected with close to equal coverage between the two datasets. This was carried out by first removing individuals from the global collection with unusually high (>97%) missing data over all unfiltered SNPs, then selecting those SNPs which had >80% coverage of individuals in the global collection before the final filtering step using the thresholds given above.

A separate SNP filtration step was performed from raw SNPs using the same process to assess the four major landrace subpopulations Tusta, Taviche, Costeño, and Chile de Agua. Also, the SNP filtration process was performed separately with and without the incorporation of the global collection, to have a set of high‐coverage SNPs for higher‐resolution genomic analyses of a subset of the accessions.

### 2.6 GBS alignments to the pepper reference genomes

To compare the GBS information with the three reference genomes, alignments of 150‐bp sequences around SNPs to the Zunla and Chiltepin reference genomes were performed. First, we extracted a 150‐bp sequence around each SNP in the CM334 reference genome. These sequences were combined in a FASTA file, which was then aligned to the Zunla and Chiltepin reference genomes using Bowtie2 (Langmead & Salzberg, 2012). The SNP genotype was taken from the base call at the Zunla or Chiltepin position aligning to the SNP position in the 150‐bp CM334 sequence.

### 2.7 Accession quality control

Three Mexican accessions were removed from the dataset by applying the following criteria. First, individuals could have no more than 30% missing data across all filtered SNPs (returned by VCFTools). Second, accessions could not cluster away from all other plants of the same named type in the initial clustering analysis (below) and be identified as distinct based on fruit phenotype. Individuals #167–1 and #218–1 were removed via the first criterion, and both plants grown from one accession (#122–1 and #122–2) were removed via the second criterion. As #167–1 was the only representative which germinated from accession 167, this quality control left 101 accessions remaining of the original 103. Forest or backyard‐grown accessions such as the one guajillo (Supporting Information Table S1) with ambiguous species characteristics that were recorded as *C. annuum* in the field, but grouped together with *C. frutescens*, were reassigned as *C. frutescens* and as such excluded from the in‐depth subpopulation structure analysis.

The two‐step SNP filtering (described in SNP‐calling above) for the combined Mexican and global collection dataset made a two‐step filtration of individuals necessary, to avoid biasing SNPs toward those covering individuals that would be later removed. Thus, prior to the first step of SNP filtering, individuals with unusually high (>97%) missing data among raw SNPs were removed from the global collection. Otherwise, filtration of individuals occurred as described above for the combined dataset.

### 2.8 Population structure

The GBS data from the Mexican collection were used to obtain a population tree. The initial tree was selected using the maximum parsimony method, followed by maximum‐likelihood optimization based on the general time‐reversible model, with 1,000 bootstraps as implemented in RAxML (Stamatakis, 2014; Tavaré, 1986). The *C. chinense* accession 155–1 was used as a midpoint to root the tree. This tree was used to filter individuals and assign individuals to preliminary groups based on their named types, locations, and genetic relatedness as revealed by clustering analysis. We also performed this analysis using less computationally intensive parameters: creating a neighbor‐joining initial tree, followed by maximum‐likelihood optimization with the Tamura‐Nei mutation model (Tamura & Nei, 1993; Tamura et al., 2011) and 100 bootstrap replicates (Supporting Information Figure S1). Finding no substantial differences between the clusters in each analysis, we used the less computationally intensive analysis to explore additional subsets of the data. Population trees were also created separately for each of the four main cultivated Oaxacan subpopulations: Tusta, Taviche, Costeño, and Chile de Agua. In addition, genetic assignment analysis was conducted using the program fastSTRUCTURE (Raj, Stephens, & Pritchard, 2014), first, with only the Mexican accessions, then with the combined Mexican and global collection dataset, including the available reference genomes. In both cases, the number of genetic clusters (*K*) was allowed to vary from 2 to 10. Accessions were assigned to the groups corresponding with their locally known types except where both clustering analysis and genetic assignment analysis assigned an accession to a group other than the named type, with a threshold of 70% identity in the latter analysis. For the cultivated *C. annuum*, five of the 80 analyzed accessions were reassigned in this way, all of which were locally known as Tusta or Taviche. Mean imputation followed by principal components analysis was performed using the package SNPRelate (Zheng et al., 2012) on the complete population, including global collection and reference genomes.

Genome scans for population origin, selection sweeps, and diversity were, respectively, performed using corrected Wright's *F*_ST_ (Weir & Cockerham, 1984), Tajima's D (calculated over segregating sites) and the pairwise nucleotide diversity measure π (measured on a per‐nucleotide basis calculated using the proportion of the genome included by the GBS reduced‐genome methodology), as implemented in VCFTools and performed only on Mexican landraces. Pairwise permutation tests were performed by shuffling individuals among population pairs, while keeping population sizes constant in R. The resulting permutated populations were saved into population files for use in VCFTools.

As an internal control and to explore diversity within each accession, two plants were grown for each accession. In all accessions for which genotypes could be recovered from both plants, both plants exhibited the same group membership pattern. Close relatedness was also demonstrated between individuals of the same seedlot, named type, and species (Supporting Information Figure S2).

## 3 RESULTS

### 3.1 Distribution and morphology of pepper types

To explore the genetic diversity of landrace and ancestral chile peppers primarily in Oaxaca, Mexico, we assembled a collection to cover diverse pepper use‐types, as well as different degrees of domestication. Several of the collected Oaxacan landraces were endemic to specific subregions (Figure 1). We collected populations of Chile de Agua (Supporting Information Table S1), a major cultivated landrace, only from the high central valleys of Oaxaca. A less cultivated, but still important, landrace population is Costeño, which we collected along the southern coastline of Oaxaca. Tusta accessions were collected from several sites along the north–south transect between the central valleys and southern coast. Taviche were collected only from San Pablo Coatlán, in the central valleys of Oaxaca. A few accessions were collected from the Yucatán and they were Maax'ik and Dulce. Some backyard‐grown named types were eclectic collections of peppers that spanned multiple species. Peppers called Paradito were diverse, with accessions spanning both the Oaxacan and Yucatán collection regions and both *C. frutescens* and *C. annuum* species (Supporting Information Table S1).

**Figure 2:**
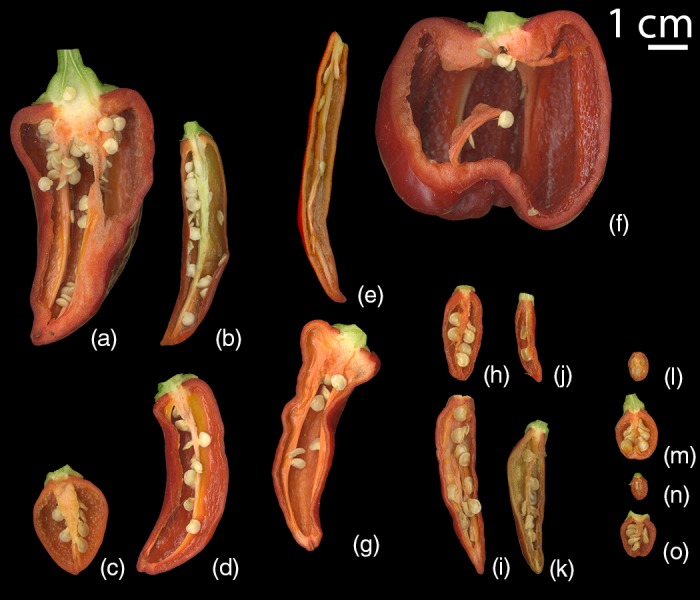
Typical fruit morphology of pepper types displayed by longitudinal scan of a single fruit. The four major types that predominated this study are on the left. Types are labeled: (a) Chile de Agua, (b) Costeño, (c) Tusta, (d) Taviche, (e) De Arbol, (f) Dulce, (g) Guiña Dahni, (h) Mirasol, (i) Solterito, (j) Mareño, (k) Piquin, (l) Paradito, (m) Chigole, (n) Bolita, (o) Payaso

The Mexican landrace populations presented a diverse set of fruit phenotypes, ranging from small and round to very narrow, and from elongated to blocky (Figure 2). The most intensely cultivated accessions (grown exclusively in *milpas* or on plantations) tended to have larger fruits (Supporting Information Table S1). These included the Chile de Agua (Figure 2a), a fresh use‐type that was grown most commonly in plantations; Costeño (Figure 2b), a dry use‐type that was grown in plantations and *milpas*; Taviche (Figure 2d), a dry use‐type collected only from a *milpa*; Guiña Dahni (Figure 2g), a dry use‐type collected from a coastal plantation in Oaxaca; and Dulce (Figure 2f), a fresh use‐type which was grown in several Yucatán *milpas*. The Mexican collection also included chile peppers more commonly grown in backyard or “let‐stand” environments, such as the De Arbol, Tusta, Mirasol, Solterito, Mareño, Piquin, Paradito, Chigole, Bolita, and Payaso (Figure 2e,c,h‐o, respectively). At last, the Mexican collection included a number of populations growing in forests and uncultivated environments, including some Chigole peppers and those colloquially known as Chile de Monte (any uncultivated peppers growing in mountains or forests). These forest‐grown chile peppers were much smaller than the cultivated types, but had seeds which were still similar in size to the cultivated types. Thus, the pericarp around the forest‐grown pepper types was little more than a thin coating around the seeds, in contrast to many of the thicker‐fleshed cultivated types. In total, the Mexican collection included 19 named types of chile peppers.

Fruit morphologies varied between different named types. All Chile de Agua plants bore fruits with a similar triangular shape with large truncate shoulders tapering into a distal point that was blunt to slightly sunken (IPGRI, 1995). All Costeño plants bore smaller, more elongate, often curved fruits with a pointed distal end (IPGRI, 1995). Accessions bearing the Tusta label (including two from San Pablo Coatlán) were heart‐shaped fruits, tending to have high shoulders above a noticeable proximal indentation, while Taviche fruits (including two that fell genetically within the Tusta subpopulation) were more similar to Costeño in size, although they tended toward wider shoulders, making them more triangular than horn‐shaped (UPOV, 2004). Both fruit phenotypes and named types from Yucatán accessions were varied. Semi‐wild peppers (a pepper where it is unclear if it is a truly wild or a feral) in both *C. annuum* and all *C. frutescens* were much smaller and tended to be rounder than the four main types: Tusta, Taviche, Costeño, and Chile de Agua, as well as the named types with only 1–2 accessions in our Mexican collection: Dulce, Guiña Dahni, and De Arbol (Supporting Information Table S1).

### 3.2 Genetic structure of Mexican chile pepper population

To describe the genetic structure of the Mexican population, we generated a GBS SNP dataset. After filtering, 32,623 SNPs were called among the Mexican accessions, and 3,570 had sufficient coverage for comparisons to the accessions from outside Central America (Supporting Information Figure S3). SNPs called by GBS were distributed mostly in the euchromatic regions, with relatively few being found in the pericentromeric regions as defined by the reference genome (Qin et al., 2014).

**Figure 3:**
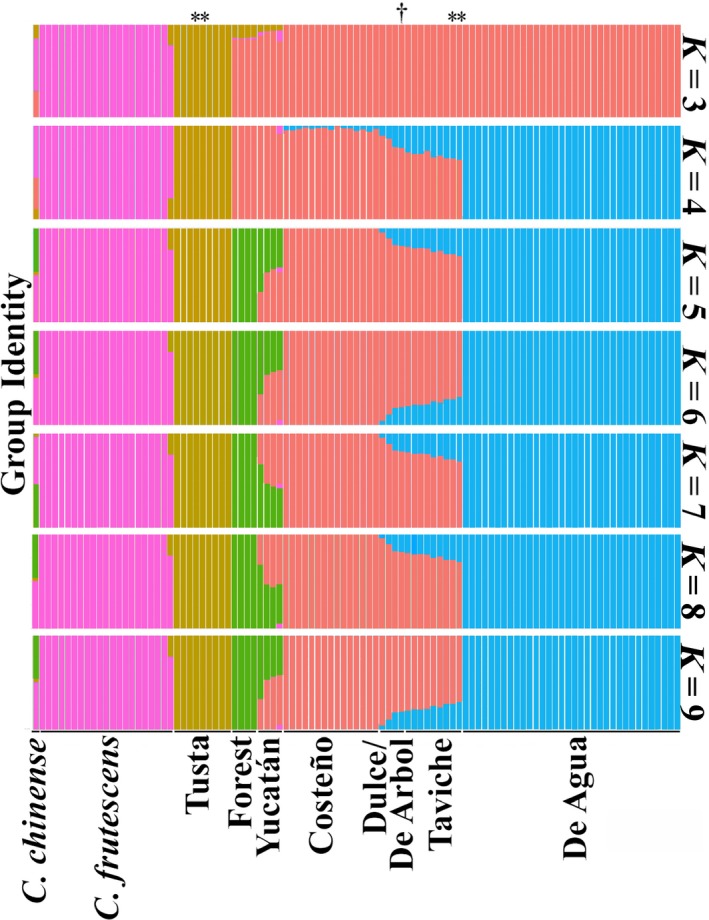
Genetic assignment plot. Depicts identity in one of *K* individually colored groups as a stacked barplot, where *K* varies from 3 to 9. The height of each bar indicates probability of membership for each of the 2013 collection accessions, laid out along the *x*‐axis. Clear differences are apparent between named landrace types. After the *Capsicum chinense* accessions, the Costeño and De Agua accessions showed the strongest single‐population membership, which is consistent with their being the largest and most restrictively cultivated populations in our collection. Asterisks indicate the Taviche and Tusta accessions bearing high identity with the Tusta and Taviche groups, respectively. Dagger indicates De Arbol accession

**Figure 4:**
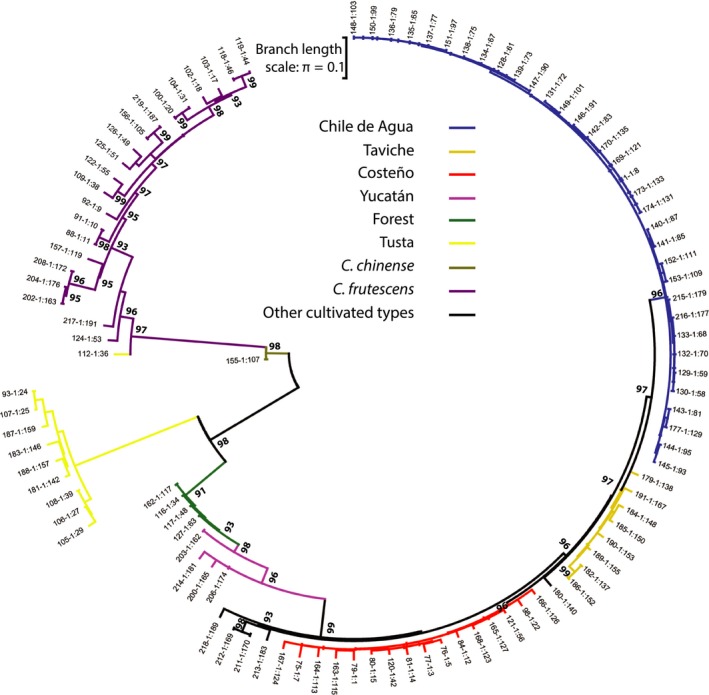
Maximum‐likelihood population tree with branches color‐coded by group. The first clade (shown in purple) included all individuals identified as *Capsicum frutescens*. The second included the Tusta landraces (yellow), and the third contained all other *Capsicum annuum* landraces. This clade also included nearly all accessions from the Yucatán, and many semi‐wild accessions collected from forest understories. Both the backyard Yucatán and forest‐collected members of the *C. annuum* clade were basal to the other members of this clade. Branch lengths (scale bar shown at top of circle) indicate average pairwise substitutions per site from last common ancestor

Using FastSTRUCTURE (Raj et al., 2014) to assess integrity and admixture in named Mexican landraces, we examined the assignment pattern with the number of subpopulations (*K*) from *K = *3–9 (Figure 3). The optimal Δ*K* (Evanno, Regnaut, & Goudet, 2005) value was predicted to be 7 (Figure 3; Supporting Information Figure S4). At *K *=* *3, there was clear differentiation between *Capsicum frutescens* and Tusta accessions, and the remaining *C. annuum* accessions. At *K *=* *4, Chile de Agua accessions were a distinct cluster. At *K *=* *5, the forest accessions were a distinct cluster. At *K *=* *5, the single *C. chinense* accession, a Maax'ik accession from the Yucatán, demonstrated admixture between the *C. frutescens* and *C. annuum* accessions. *C. annuum* accessions within the same named type exhibited similarity in the genetic assignment analysis. As demonstrated by the structure plots for *K = *6 through *K *=* *9, higher levels of *K* created superfluous groups explaining very little variation (Figure 3; Supporting Information Table S2). In summary, the analysis identified three main subpopulations among the cultivated accessions, Tusta, Costeno, and Chile de Agua. Taviche accessions did not represent a separate group in the genetic assignment analysis, but instead shared a pattern of admixture between Chile de Agua (~25%) and Costeno (~75%). Based on this, their local names, and their significant bootstrap value in the population tree analysis we show below (Figure 4), we found it useful to analyze Taviche separately.

Both plants from Tusta‐type accessions (#179 and #185, Supporting Information Table S1) grown in San Pablo Coatlán alongside a Taviche population (indicated by stars Figure 3) showed a genetic subpopulation identity pattern that was indistinguishable from peppers in the Taviche subpopulation, and showed no membership in the Tusta subpopulation. All Tusta and Taviche types grown from seed in our greenhouses were phenotypically distinct, each bearing fruits characteristic of their respective parental named types (Figure 2). Therefore, despite being morphologically similar to Tusta, these San Pablo Coatlán “Tusta” were genetically more closely related to the Taviche than to Tusta taken from Santa Lucia Miahuatlan (#188), San Baltazar Loxicha (#187), Los Reyes (#105–#108), or Juan Diegal (#93). These accessions were henceforth considered part of the Taviche subpopulation rather than the Tusta subpopulation. Likewise, two Taviche‐named accessions (#181 and #183; Supporting Information Table S1) were closely related to the Tusta (indicated by stars above the corresponding bars in the Tusta subpopulation in Figure 3). In all four cases, the two plants derived from each accession (#179, #185, #181, and #183) were paired in clustering (Supporting Information Figure S2), indicating that a DNA mix‐up was unlikely and would have required mistakes to have occurred independently in the handling of both plants from each accession. Confirming that the fruits collected from each plant matched the parental type of the accession from which it was grown similarly excluded a seed mix‐up (Supporting Information Table S1).

The single de Arból accession appeared genetically in between Costeño and Taviche. Membership in the remaining subpopulations was distributed among two types of accessions: (a) those that were considered “semi‐wild” accessions of *C. annuum* collected from the forest understory or backyards known as Chigole or Chile de Monte, and (b) the less restrictively cultivated accessions from Yucatán (belonging to Maax'ik and Xaat'ik) sharing genetic diversity with the semi‐wild and Costeño.

To relate the accessions to one another, we reconstructed a population tree of the Mexican chile pepper collection rooted at the midpoint by the single *C. chinense* accession (Figure 4). We detected three main groups with high (>95%) bootstrap support that agreed with our previous population assignments from structure. Except for the *C. frutescens*‐like Paradito population and the Dulce accession, the Yucatán accessions clustered into basal clades that were sister to the domesticated *C. annuum* (Figures 3 and 4). Small‐fruited accessions grown in the Yucatán such as the Maax'ik and Paradito were closely related to the *C. annuum* accessions grown in backyards in the southern tip of the Oaxacan coast. The accessions within the main fresh and dry use‐types in this study, Chile de Agua and Costeño, respectively, formed separate clusters (Figure 4).

We further characterized the spatial distribution of genetic diversity within the four main Mexican *C. annuum* landraces (Tusta, Taviche, Chile de Agua, and Costeño). Chile de Agua was only collected in the central valley of Oaxaca. Yet, the various Chile de Agua populations appeared to have retained interpopulation spatial differentiation. Proceeding clockwise from the top of the population tree for Chile de Agua, the most highly domesticated landrace we collected (Supporting Information Figure S5A), the first clade (labeled “i”) contained six accessions from two sites on the eastern side of the high central valley's: La Labor and Paraje Coatequillas. These sites were also connected by Federal Highway 175 (Supporting Information Figure S6). The next two clades (ii–iii) were both composed of individuals from southeastern Paraje Coatequillas. Continuing clockwise, the next clade (iv) was composed of two individuals from a northwestern collection site in La Lobera (ID #140, #141, Supporting Information Table S1). Four more accessions from a southern site—Coatecas Altas, near Paraje Coatequillas—formed a fifth clade (v). Accessions from two western sites—southwestern Santa Cruz Nexila (#1, #169, #170, #173, #174) and northwestern La Lobera (#142)—formed a sixth clade (vi), with somewhat weaker bootstrap support and the five Santa Cruz Nexila accessions showing little genetic diversity between them. A seventh clade (vii) was composed of four accessions from the eastern sites La Labor and Coatecas Altas. An eighth clade (viii) was comprised of accessions from western sites Santa Cruz Nexila and La Lobera. Thus, clades vi and viii were both distributed among collection sites in the eastern side of the Oaxacan Central Valleys, connected by highway 131 (Supporting Information Figure S6), whereas clades i and vii were distributed among western collection sites.

Costeño included more backyard accessions than the mostly plantation‐grown Chile de Agua and showed less evidence of subclades (Supporting Information Figure S5B). The exception to this is the first four accessions (ix), which all were taken from the village of Rosedalito near the southern tip of the Oaxacan coast and did constitute a clade. Beyond that, however, there was evidence for admixture, with genetic diversity apportioned more strongly between individual accessions within the same site, and only weak evidence (≤70% bootstrap) of subclades within the Costeño.

Genetic diversity was lowest within Costeño and Chile de Agua landraces compared to the other *C. annuum* landraces (Table 1). As defined after our genetic structure analysis, the Tusta and Taviche groups each formed a monophyletic clade (Figure 4). One clade comprised Taviche accessions from San Pablo Coatlán and Ejutla de Crespo (Supporting Information Figure S5C), while the other included a mixture of Tusta accessions from various sites (Supporting Information Figure S5D).

**Table 1:** Within‐population diversity for main *Capsicum annuum* landraces

Group	*n*	SNPs	π	Within‐accession	Filter Threshold for Heterozygosity (%)
				IBS (%)	
Taviche	8	4,056	0.00349	96.8% A (*S*D = 0.8%)	5%
Costeño	15	3,355	0.00222	96.9% A (*SD* = 1.1%)	2.5%
Tusta	9	9,659	0.00538	98.7% B (*SD* = 0.9%)	5%
Chile de Agua	34	7,403	0.00144	99.6% C (*SD* = 0.4%)	2.5%

Note

Results of re‐filtering SNPs within the main *C. annuum* landraces included in our Mexican collection. Column *n:* number of accessions within each landrace subpopulation; π*:* average pairwise differences per nucleotide; *% IBS:* within‐accession identity‐by‐state (group average using Mexican collection‐wide SNPs) with different letters indicating significantly different groups. The last column gives maximum allowed heterozygosity for each SNP during filtering.

### 3.3 Allele frequency differentiation and genetic diversity

The average nucleotide diversity (π) within each use‐type group ranged from 0.031% to 0.01% and appeared to decrease with intensity of cultivation (high cultivation for Chile de Agua and low cultivation for Tusta) (Table 1). Despite containing the fewest accessions, the Tusta group had the most segregating SNP variation and the highest π. Chile de Agua contained the second‐highest number of segregating SNPs. Despite that, Chile de Agua showed the lowest π of all four main groups, indicating a high degree of homogeneity within the accessions. Homogeneity was also evaluated as the percent identity‐by‐state (% IBS) between same‐accession pairs (two plants per seedlot), calculated over all nonmissing loci for each accession, and averaged over all accessions within each group. The within‐accession average IBS percentage was high in each of the four groups, relative to the overall mean IBS of 72% (*SD* = 22%). Percent IBS differed significantly between groups (*F *= 57.56_3,7_, *p *<* *0.001). Post hoc comparisons using a Bonferroni‐corrected LSD test indicated that the Chile de Agua accessions were significantly more homogenous, and the Taviche and Costeño were significantly less homogenous than Tusta as measured by within‐accession average percent IBS (Table 1).

As admixture was apparent between certain subpopulations in our collection, we used *F*_ST_ to quantify the genetic distance between the admixed populations. Mean *F*_ST_ averaged over all cultivated landrace populations was 0.821 after correcting for population size and substructure. The highest pairwise *F*_ST_ was between the Chile de Agua and the Tusta landrace (Table 2). Among the *C. annuum* accessions we studied, the allele frequencies of the Tusta landrace were most distinct from those of the other cultivated *C. annuum* landraces (Table 2). Pairwise comparisons involving the *C. frutescens* clade, which we defined as spanning all individuals identified as *C. frutescens* based on morphological observations*,* and the population of individuals unambiguously belonging to *C. annuum* gave consistently high *F*_ST_ values (0.667–0.892), as did comparisons between Tusta and any other subpopulation (Table 2). Pairwise *F*_ST_ demonstrated that all named types were significantly distinct from each other in terms of allele frequencies (*p* < 0.001, permutation test). Pairwise *F*_ST_ analysis also revealed that Taviche, Costeño, and Chile de Agua were more closely related to each other than to Tusta or to the *C. frutescens* subpopulation (Table 2), recapitulating the pattern previously revealed (Supporting Information Figure S2).

**Table 2:** Pairwise, corrected *F*_ST_ values for major population pairs

	*Capsicum frutescens*	*Capsicum annuum*	Forest	Taviche	Tusta	Costeño	De Agua
*C. frutescens*	‐	0.74231	0.66724	0.75646	0.81053	0.82345	0.89223
*C. annuum*	***	‐	NA	NA	NA	NA	NA
Forest		NA	‐	0.19887	0.75809	0.42406	0.59077
Taviche	***	NA		‐	0.71391	0.21242	0.46114
Tusta	***	NA		***	‐	0.87761	0.95156
Costeño	***	NA		***	***	‐	0.63705
De Agua	***	NA		***	***	***	‐

Note

Values above the diagonal are average *F*_ST_ values for all SNPs calculated using Weir and Cockerham's corrected *F*_ST_ (1984). Below the diagonal, levels of significance are indicated. NAs indicate pairwise *F*_ST_ not applicable because populations are nested. All pairs had *F*_ST_ values significantly higher than admixture (*** indicates *p* < 0.001, permutation test of 10,000 permutations).

### 3.4 Genome scans for selective sweeps

Identifying the Chile de Agua and Costeño as clearly distinct populations allowed us to analyze each of these populations for genomic statistics of diversity. Therefore, we assessed which genomic regions might diverge from neutral evolution for further study of adaptations that are specific to those landraces. We determined that a bin size of 500 kb was reasonable by linkage disequilibrium (LD) decay analysis (Supporting Information Figure S7), and focused on the SNP‐rich euchromatic regions. Several regions in the Chile de Agua genome appeared to exhibit clusters of extreme Tajima's D values for nearby bins (Supporting Information Figure S8), including an extreme low cluster at about 225 Mb on the CM334 reference genome chromosome 6 assembly (Kim et al., 2014) (Supporting Information Figure S8). Low Tajima's D values would indicate that minor alleles in a genomic region were rarer than would be expected in a neutrally evolving population, possibly suggesting the presence of a gene or genes under strong purifying selection at the bottom of chromosome 6. The lack of a corresponding low‐diversity region in Costeño suggests this as a potential candidate region for genes that control either Chile de Agua's fresh use type phenotype or local adaptation.

### 3.5 Genetic comparisons between the Mexican and Global Chile pepper populations

**Figure 5:**
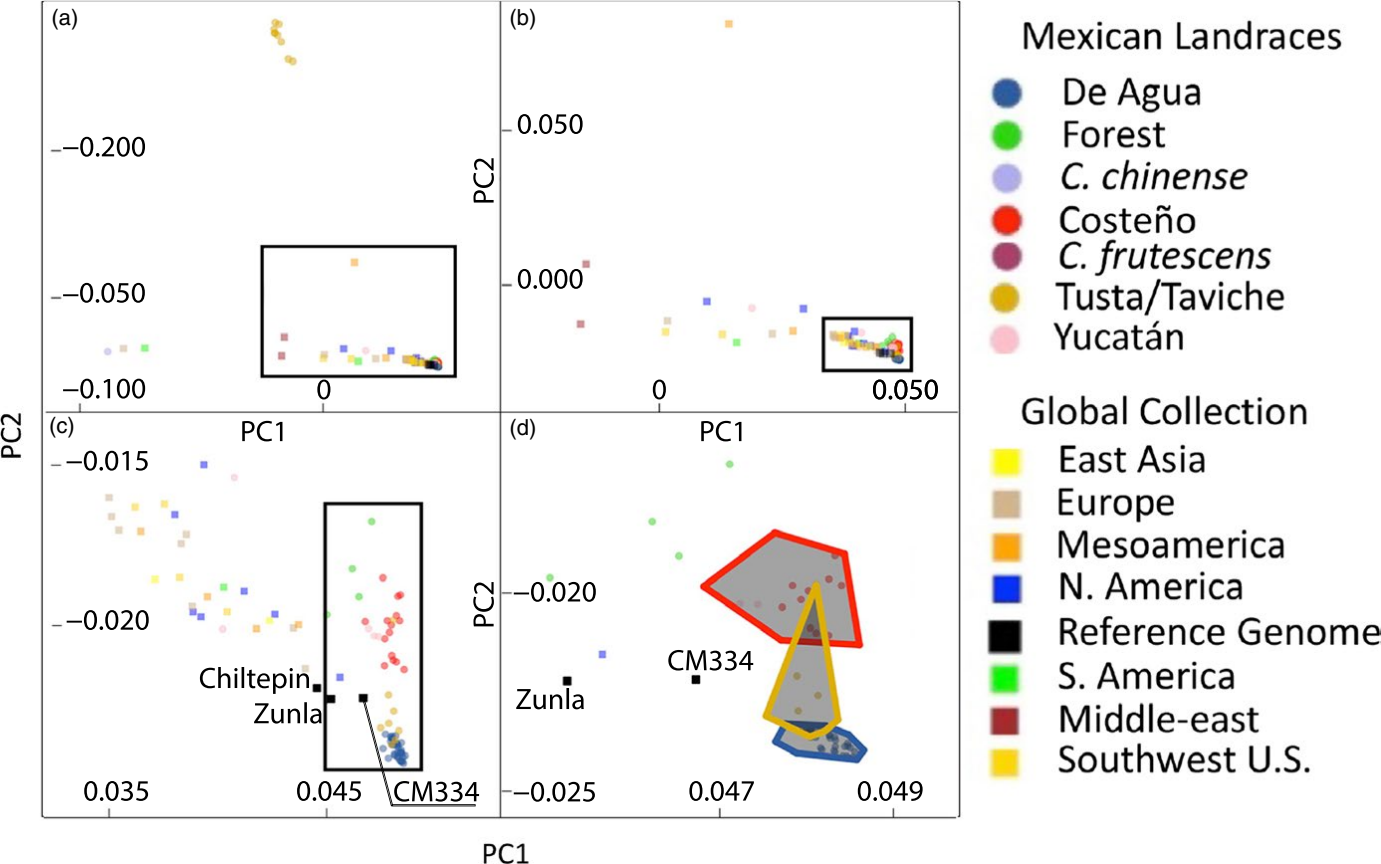
Principal components analysis showing genetic structure. Computed over all collections and the three published reference genomes. Gray frames in (a, b, and c) show the area of extent for the subsequent‐lettered panel. Members of our population grown from the 2013 Mexican collection trip are indicated by colored circles, members of our global collection set are indicated by colored squares, and the published reference genomes are indicated by dark gray squares. Oaxacan landraces grouped together, including the Criollo de Morelos 334 (CM334) reference genome. Several Yucatán accessions clustered away, near the global set

To compare the Mexican chile pepper accessions with a subset of globally grown accessions, we selected 3,570 SNPs with sufficient coverage in both datasets to make comparisons. A principal component analysis (PCA) showed that the Tusta population maintained its distinctive separate clustering pattern and represented a unique portion of the genetic diversity in *C. annuum* (Figure 5a). Looking closer at the main *C. annuum* group (excluding Tusta), the relatively greater spread of the 32 global *C. annuum* accessions indicated a greater degree of genetic diversity than among our main group of Mexican *C. annuum* accessions (Figure 5b). Focusing in on the main group of Mexican accessions and those global accessions clustering closest with them, we observed that the two published reference genomes from accessions Zunla (Qin et al., 2014) and CM334 (Kim et al., 2014) tended to group nearer to the Mexican accessions than those of the global collection (Figure 5c). Oaxacan landraces grouped together and encompassed the CM334 reference genome. Some overlap of Taviche with Chile de Agua and Costeño was also observed in the PCA plot (Figure 5d), the latter being consistent with the admixture shown in genetic assignment analysis.

**Figure 6:**
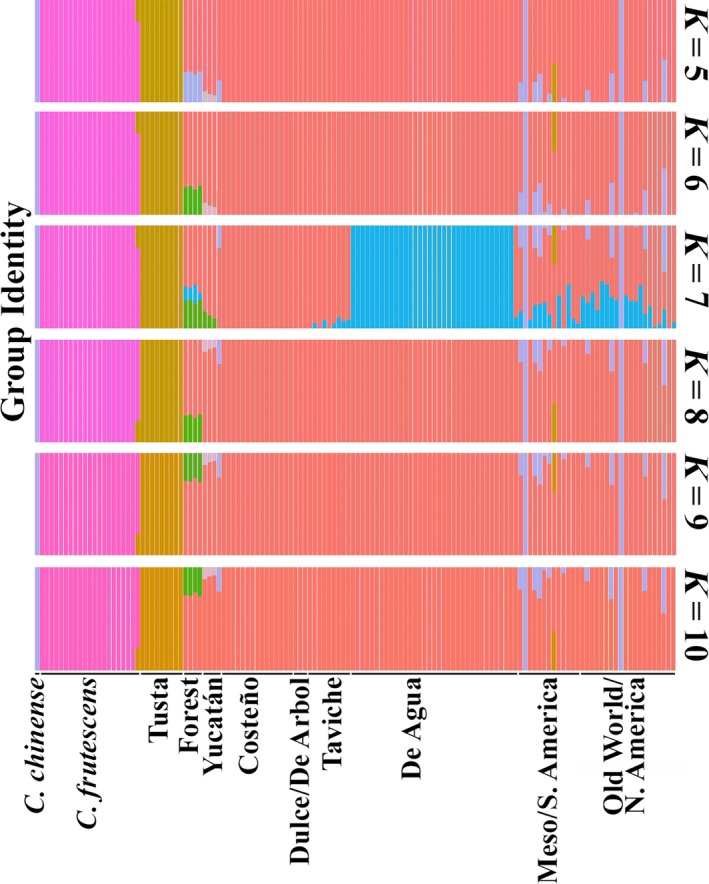
Genetic assignment plot including global collection set. Major groups within the 2013 Mexican collections were recapitulated, with the exception of the *Capsicum frutescens*, which had only one group resolved, and the Yucatán accessions, which included a unique group. The genetic structure of the global population was not clearly related to geographic origin, and *Capsicum chinense* identity appeared scattered across the global chile pepper collection

Genetic assignment analysis combining both the Mexican and global datasets using fastSTRUCTURE recapitulated the major Mexican landrace identities at an optimum *K* of 7 (Figure 6; Supporting Information Figure S9; Supporting Information Table S3). In addition, it revealed high levels of shared identity between the Costeño, Chile de Agua, and most of the accessions throughout the global collection. A *C. chinense* group was resolved by the addition of the global collection, which included inadvertently some *C. chinense* accessions. Also, partial membership to the same group as the single Mexican *C. chinense* accession was scattered throughout even the *C. annuum* global accessions (Figure 6; Supporting Information Table S3), also demonstrating the high level of genetic diversity among the global collection (Figure 5).

## 4 DISCUSSION

### 4.1 Genetic structure of the pepper collection population

This study validated the named chile pepper types in Oaxaca as genetically separate landraces that are distinct. We also found strong evidence of separation among the main landraces grown under intensive cultivation for market. Previous studies on genetic structure in chile peppers either focused on few markers in relatively densely sampled *C. annuum var glabriusculum* populations (Hernández‐Verdugo et al., 2001; González‐Jara et al., 2011) or many markers but few representatives of many widely dispersed cultivar populations (Hill et al., 2013; Hulse‐Kemp et al., 2016; Naegele et al., 2016). Such studies have found that humans are largely responsible for driving gene flow and therefore population structure in chile pepper populations (González‐Jara et al., 2011), and have found evidence that pepper cultivars grown today are descended from a few initial populations (Hulse‐Kemp et al., 2016).

By sampling multiple individuals from each of several Mexican landraces, we were able to delve into these genetic patterns differentiating the landraces. In doing so, we determined which landraces are candidates for being major contributors to many cultivars now grown around the world. We found a higher degree of genetic structure in our Mexican collection of chile peppers than had previously been reported for maize landraces grown in the same area (Pressoir & Berthaud, 2004b). This could be due to the relatively limited pollen dispersal in chile peppers (Raw, 2000) leading to greater inbreeding. Genetic assignment patterns were partitioned as expected, grouping together plants from the same species, major landrace types, and our internal biological replicate controls. *C. frutescens* and *C. chinense* were distant from each other and from the main *C. annuum* clade. Both the forest‐grown and Yucatán *C. annuum* accessions were located basal to the Mexican landraces in our population tree analysis, supporting the hypothesis that these landraces were derived in common from a broader population including both the forest and Yucatán accessions (Figure 4).

Comparing the Mexican collection to the global collection, we found evidence of admixture among the different landraces as components of the highly genetically diverse global collection, consistent with patterns of global admixture from a few initial populations, found previously (Hulse‐Kemp et al., 2016; Naegele et al., 2016). Comparing these two datasets to the two independently published reference genomes CM334 and Zunla, we found that the CM334 assembly, created from the Mexican landrace with polygenic resistance to the oomycete pathogen *Phythophthora capsici* (Ogundiwin et al., 2005), clustered more closely with the Mexican collection in principal components analysis (Figure 5). Further research, exploring whether some resistance QTLs are shared between CM334 and these or other Mexican landrace, seems promising.

### 4.2 Levels of genetic diversity differed among landraces at various scales

Our study emphasized the four named types of *C. annuum* grown most commonly in Oaxaca: Chile de Agua, Costeño, Tusta, and Taviche. The first two populations were grown almost exclusively in the most restrictively managed plantation environments, whereas the latter two were found in the less‐restrictive *milpa* polycultures or in backyards. None of the four were found growing feral in forests. Genetic assignment and clustering analyses revealed that these named types did indeed comprise four major and separate genetic subpopulations of *C. annuum*, and could be considered separate landraces. The major fresh and dry use‐types: Chile de Agua and Costeño, respectively, were validated as independent, although closely related populations. These two landrace groups show the strictest spatial distribution: No Chile de Agua in our study was collected from the low coast, and no Costeño was collected from high elevation. Separate Tusta and Taviche populations were also identified, with the exception of four accessions phenotypically resembling one type and genetically resembling the other. As might be expected from a self‐pollinating species (Loveless & Hamrick, 1984), genetic diversity was partitioned primarily among, rather than within, these four landrace types.

The Costeño and Chile de Agua landraces had lower overall diversity (π), as would be expected for a restrictively managed plantation cultivation system, in which off‐types would be removed from the population. Within single‐accession seedlots, however, we found significantly higher diversity in the Costeño than the Chile de Agua. This result is expected based on the higher degree of structure in the Chile de Agua, relative to the Costeño. Stronger genetic structure, coupled with inbreeding, is expected to depress effective population size and heterozygosity at both the individual plant and population levels (Loveless & Hamrick, 1984). Despite both landraces being found in plantations, and overall genetic diversity being similar for both, the genetic data indicate a larger degree of outcrossing among the Costeño, and more isolation between Chile de Agua subpopulations. A possible explanation for this difference in within‐accession diversity lies in the different geographic factors of each landrace's growing region. While the coastline provides a natural trade route for seed sharing among coastal villages, the mountains scattered among the central valley's where Chile de Agua are grown could have historically served as an impediment to trade among mountain villages, and to gene flow among their crop populations.

Although our population was highly structured, we did observe some admixture that can be explained by the large amount of farmers’ seed sharing followed by crossing (González‐Jara et al., 2011). The pattern shown in Chile de Agua reflects a combination of these two forces: The overall landrace population is structured into different subpopulations genetically, but several of those subpopulations are spread among multiple sites rather than being endemic to one location. Not all Chile de Agua subpopulations are present at all Central Valley sites. No subpopulation supported by a bootstrap value of greater than 90% contained two accessions from opposite (e.g., northeast and southwest) corners of the central valley. However, clustering analysis suggested that Chile de Agua populations grown in a single village were combinations of once‐separate subpopulations as several well‐supported Chile de Agua clades were spread out in the north–south direction along two highways running down each side of the central valley's (Supporting Information Figure S6).

The Tusta population in our dataset was separate from the rest of the *C. annuum* in clustering analysis. The *C. frutescens* accession with Tusta admixture in the genetic assignment analysis may offer a clue to this pattern. As Tusta were found almost exclusively in backyards, they may have a more complicated genealogy than the carefully isolated, row‐crop grown Costeño and Chile de Agua. This is consistent with a hybridization event between an ancestor of cultivated *C. annuum* Tusta ancestor and an ancestor to one of the many backyard‐grown *C. frutescens*. Such hybridization could explain the relatively large size of Tusta fruits despite its genetic location basal to the *C. annuum* in clustering analysis, including the small‐fruited forest‐growing *C. annuum*. Also, individuals with ancestry including hybridization between relatively distant lineages often cluster toward the basal parent in clustering analyses (McDade, 1992), as we see in our Tusta subpopulation. However, there was a relatively high level of within‐accession homogeneity in the Tusta in this dataset that would not be expected for segregating seeds generated from a highly heterozygous hybrid population. The isolated backyard environments in which Tusta are grown may be responsible for this homogeneity. Several generations of selfing due to such isolation would be sufficient to increase within‐accession homogeneity while maintaining the overall genetic pattern of a historic hybrid genealogy, analogous to the production of a recombinant inbred line following a test cross.

The dry‐use Taviche exhibits admixture between Costeño and Chile de Agua populations. Taviche also had the lowest within‐accession homogeneity of all landraces studied, which could be consistent with relatively recent hybridization. The high levels of heterozygosity resulting from that hybridization event would have been fortified against fixation by their pollinator‐friendly *milpa* conditions (Landaverde‐González et al., 2017), and geographic proximity to other Taviche stands. In fact, the Taviche in this collection came from just one location in San Pablo Coatlán. Furthermore, despite the fact that Tusta and Taviche types had clear morphological differences and assorted into two distinct populations in all genetic analyses, two accessions from each type displayed genetic closeness to the other. For example, two accessions displayed the morphological characteristics of Tusta, but were genetically closer to the Taviche. Also, these accessions were collected from the San Pablo Coatlán site from which our Taviche were collected. This could be the result of selection‐directed introgression of Tusta morphological traits into the Taviche background. A similar introgression pattern was observed in maize landraces in Oaxaca, where divergent selection caused phenotypic diversification despite overall genetic similarity due to continued gene flow, with genetic effects only discernable as Wahlund effects near the selected loci (Pressoir & Berthaud, 2004b, 2004b). Perhaps the pollination‐permissive *milpas* (Landaverde‐González et al., 2017) where Tusta and Taviche are grown together enhance the likelihood for these normally more selfing landraces to outcross and exhibit more maize‐like population genetic behavior.

### 4.3 Signals of selection and differentiation across the genome

Using Tajima's D, we identified a region on chromosome 6 in which the Chile de Agua showed evidence of purifying selection (Supporting Information Figure S8). This pattern may be consistent with a QTL allele—such as one conferring a preferable fresh‐use phenotype—under selection in the Chile de Agua. Several loci on chromosome 6 are associated with fruit morphology (Han et al., 2016; Hill et al., 2017) including pericarp thickness (Rao, Chaim, Borovsky, & Paran, 2003), and flowering phenology traits (Yarnes et al., 2012), all of which might be under selective pressure in Chile de Agua. While such QTL could be potentially interesting candidates for follow‐up research, a list of candidates from these data would be highly speculative and were thus not included.

## 5 CONCLUSIONS

In this study, we explored a new collection of chile peppers, which was mostly focused on diverse Mexican landraces that had distinctly different uses. We explored the genetic structure of this collection, identifying that historic use‐types formed distinct genetic groups. We found that genetic diversity appeared to be related to the cultivation techniques used for the different landraces. In one landrace cluster (Tusta), there appeared to be a historic hybridization event in an ancestor to one of the many backyard‐grown *C. frutescens*, leading to both an interesting genetic and morphological place within the collection. In addition, we identified signals of selection on chromosomal regions associated with fruit morphology. There was considerable admixture in the global collection as landrace distinction broke down with peppers grown worldwide. This information has provided several hypotheses for future work including exploring differential selection for disease resistance, abiotic stress, and understanding the fine structure of ancient hybridization.

## CONFLICT OF INTEREST

None declared.

## DATA ARCHIVING STATEMENT

Sequence data for this study are available on the National Center for Biotechnology Information repository under BioProject ID PRJNA472885 and at the following URL: http://www.ncbi.nlm.nih.gov/bioproject/472885. Genotype tables are available on the DRYAD data repository at https://doi.org/10.5061/dryad.f1782h7.
