# Supplementary figures and images for: Genome‐wide genotyping of a novel Mexican Chile Pepper collection illuminates the history of landrace differentiation after Capsicum annuum L. domestication
Source: Evol Appl. 2018 Jul 11;12(1):78–92. doi: 10.1111/eva.12651 (PMC6304684; doi:10.1111/eva.12651)

**Figure S1. High-bootstrap clustering tree.**

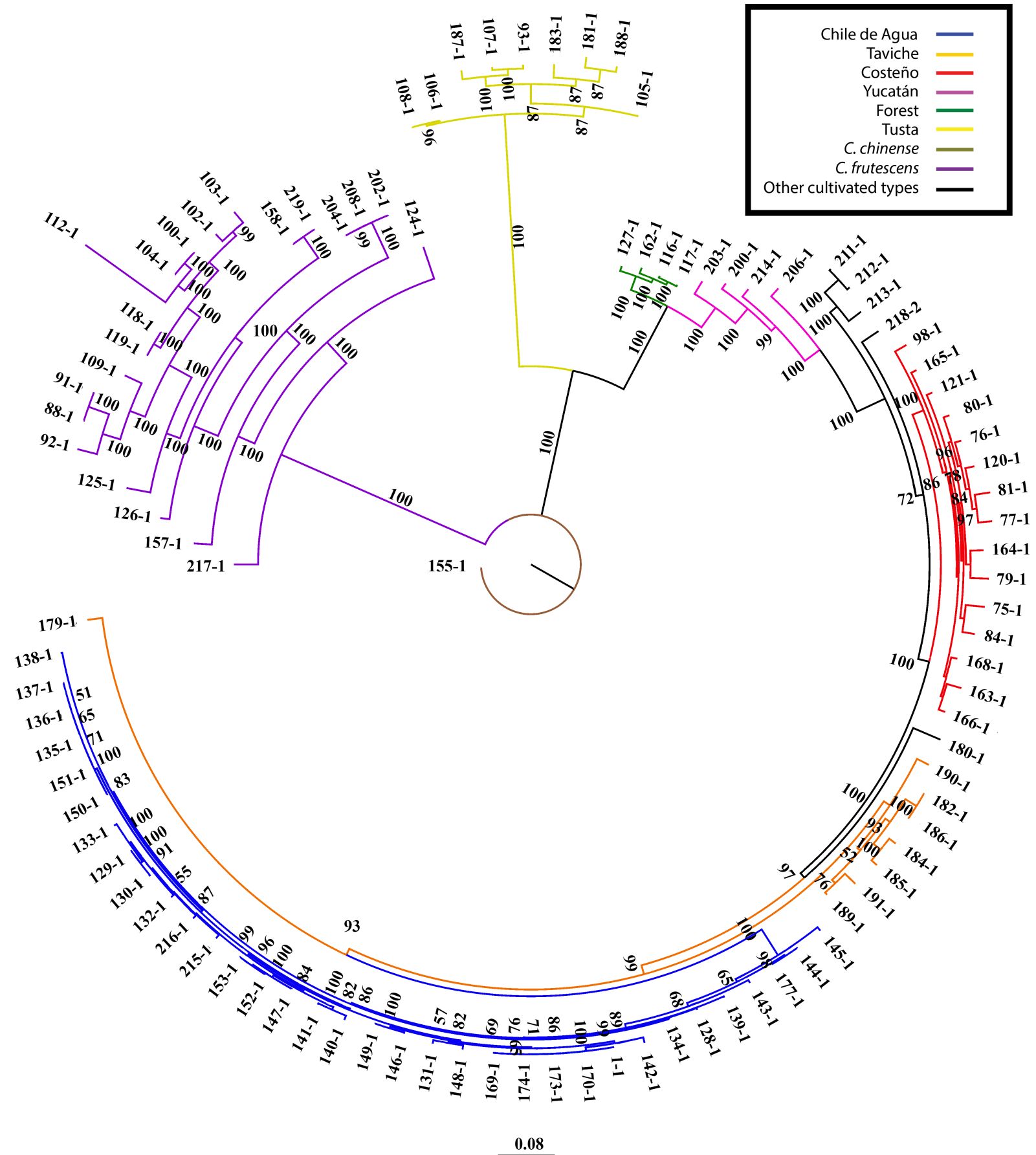

Supplement: Supplementary file 1 [file EVA-12-78-s001.pdf]

**Figure S2. Population tree (see Figure 4) including both replicate plants from each accession.**

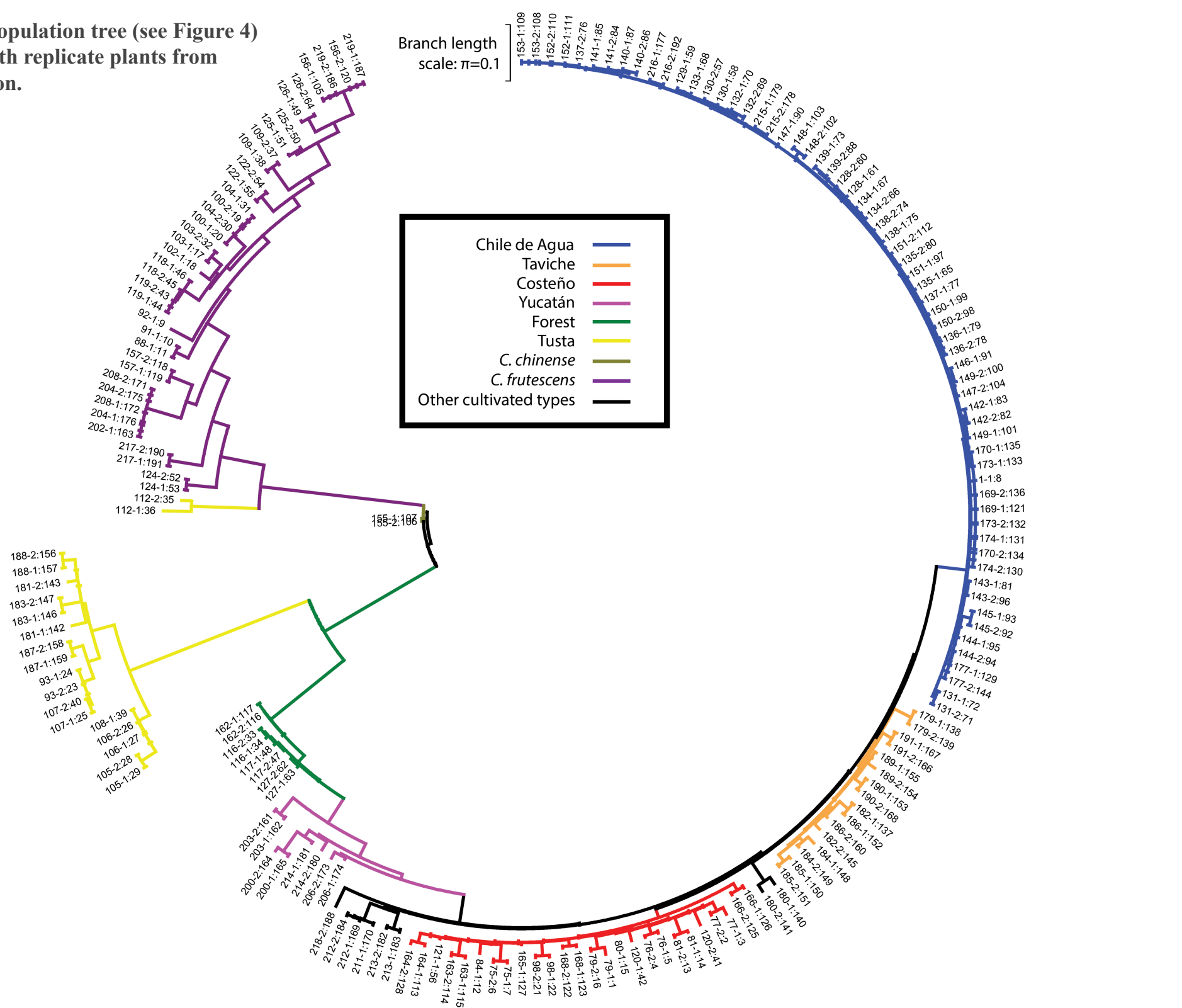

Supplement: Supplementary file 2 [file EVA-12-78-s003.pdf]

**Figure S3. Density maps showing the position of SNPs used over each pepper chromosome.**

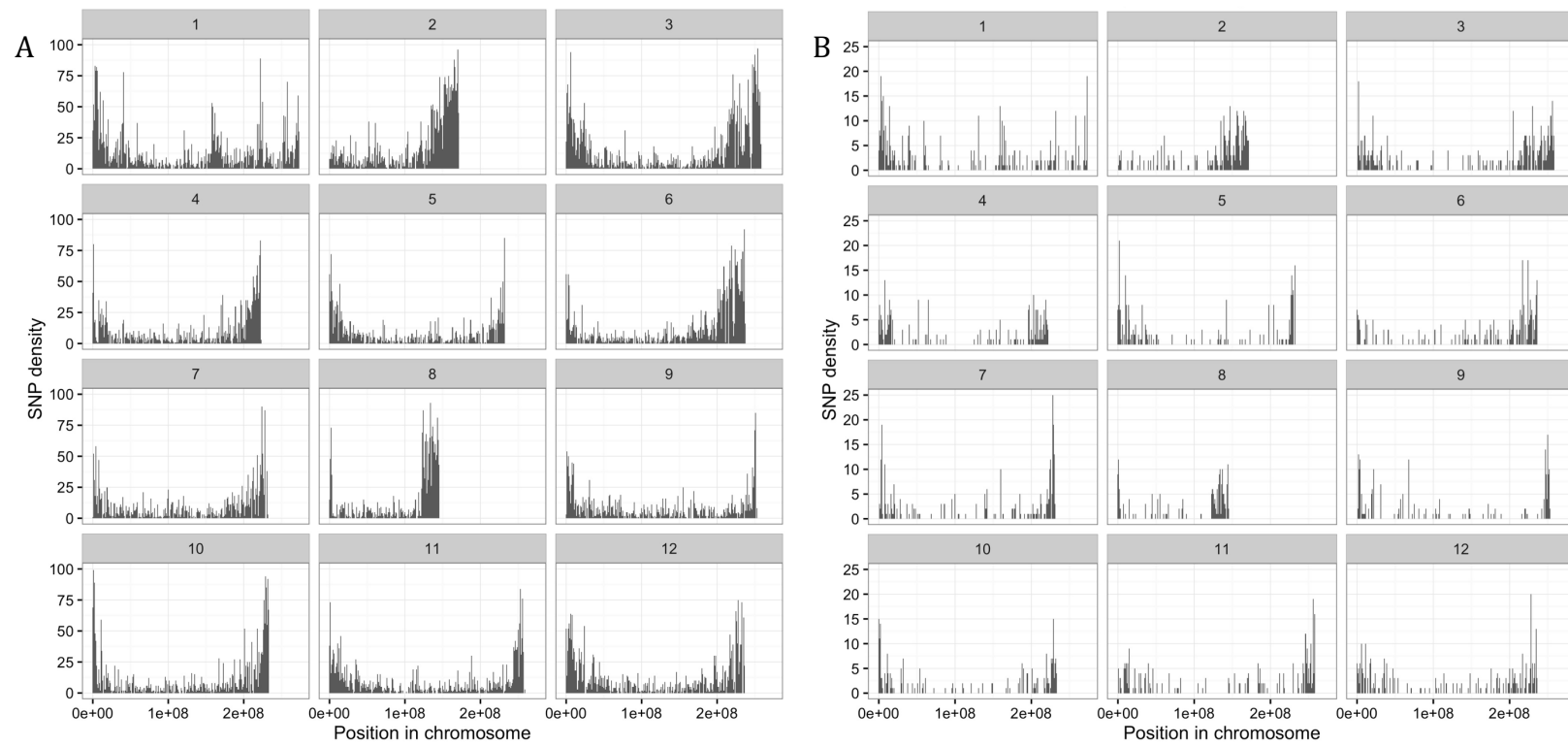

Supplement: Supplementary file 3 [file EVA-12-78-s004.pdf]

**Figure S4.  $\Delta K$  plot for Mexican peppers.**

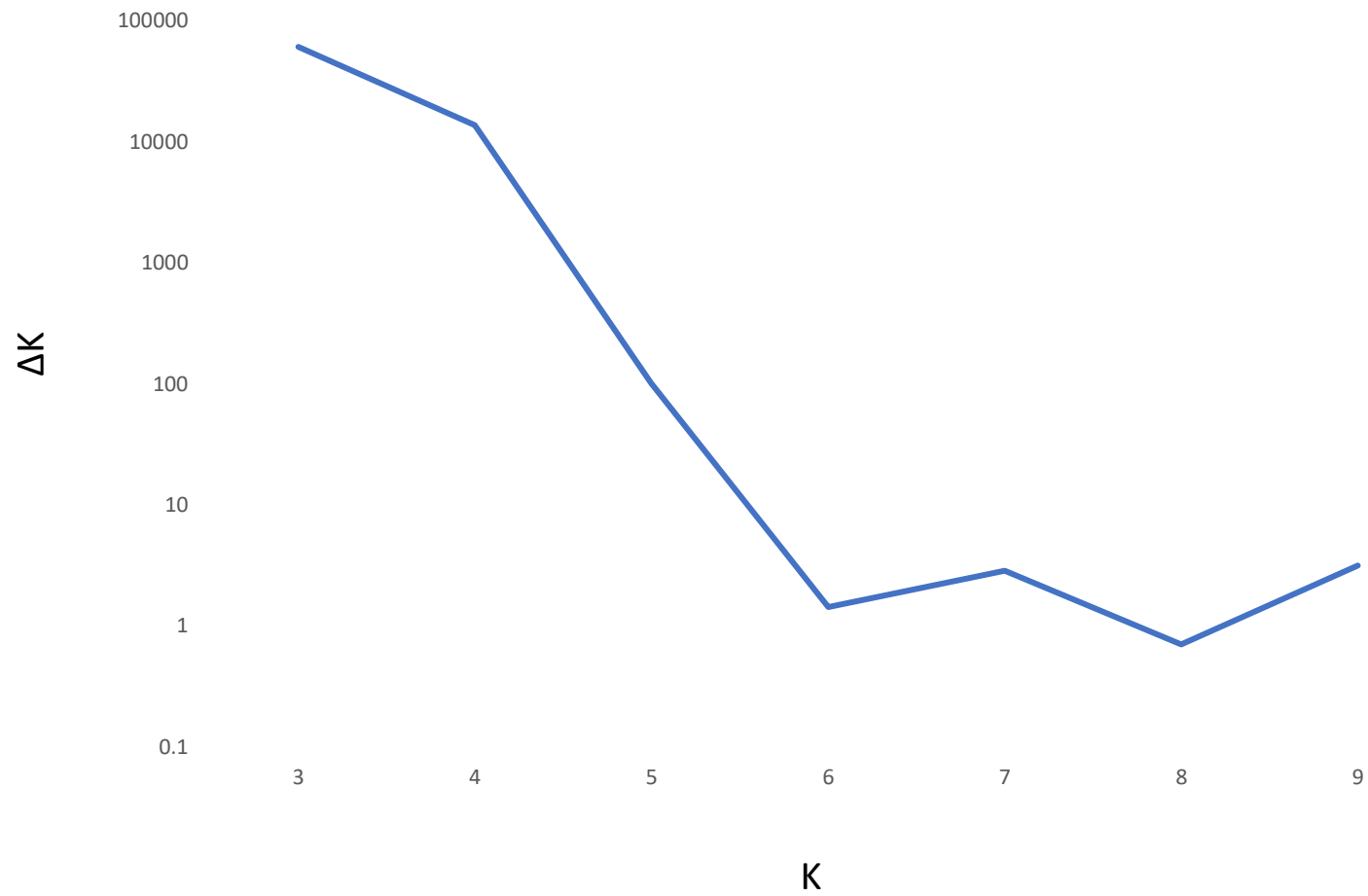

Supplement: Supplementary file 4 [file EVA-12-78-s005.pdf]

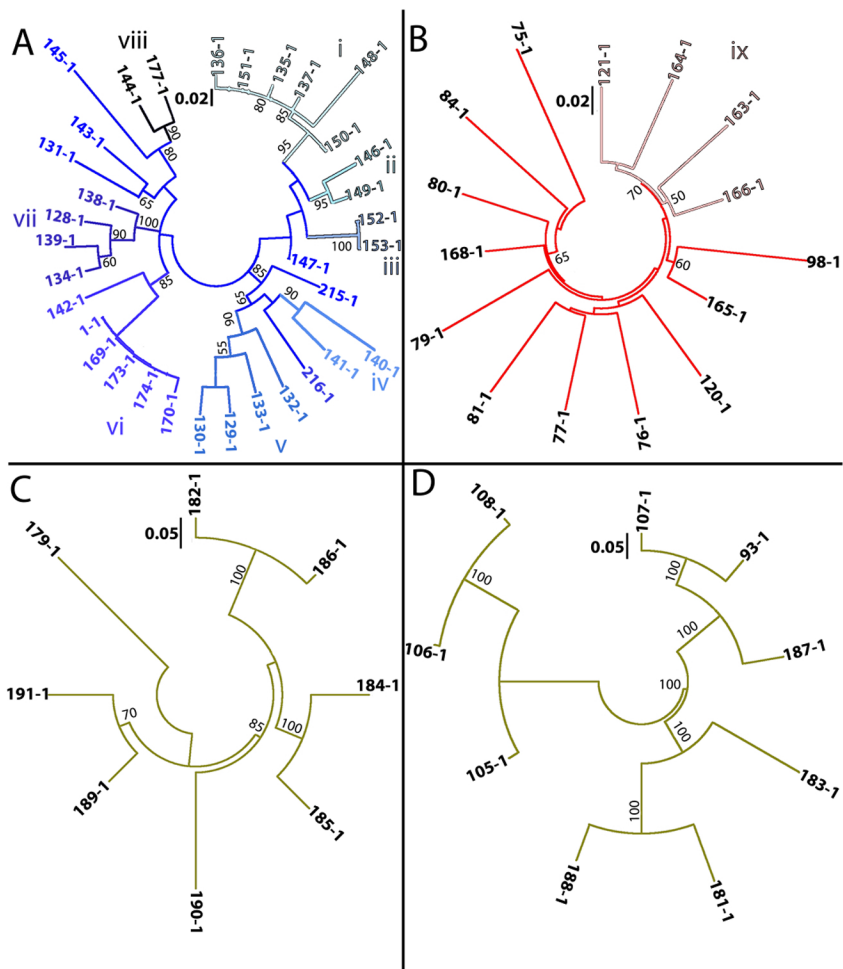

**Figure S5. Subtrees of the four major named types.**

Supplement: Supplementary file 5 [file EVA-12-78-s006.pdf]

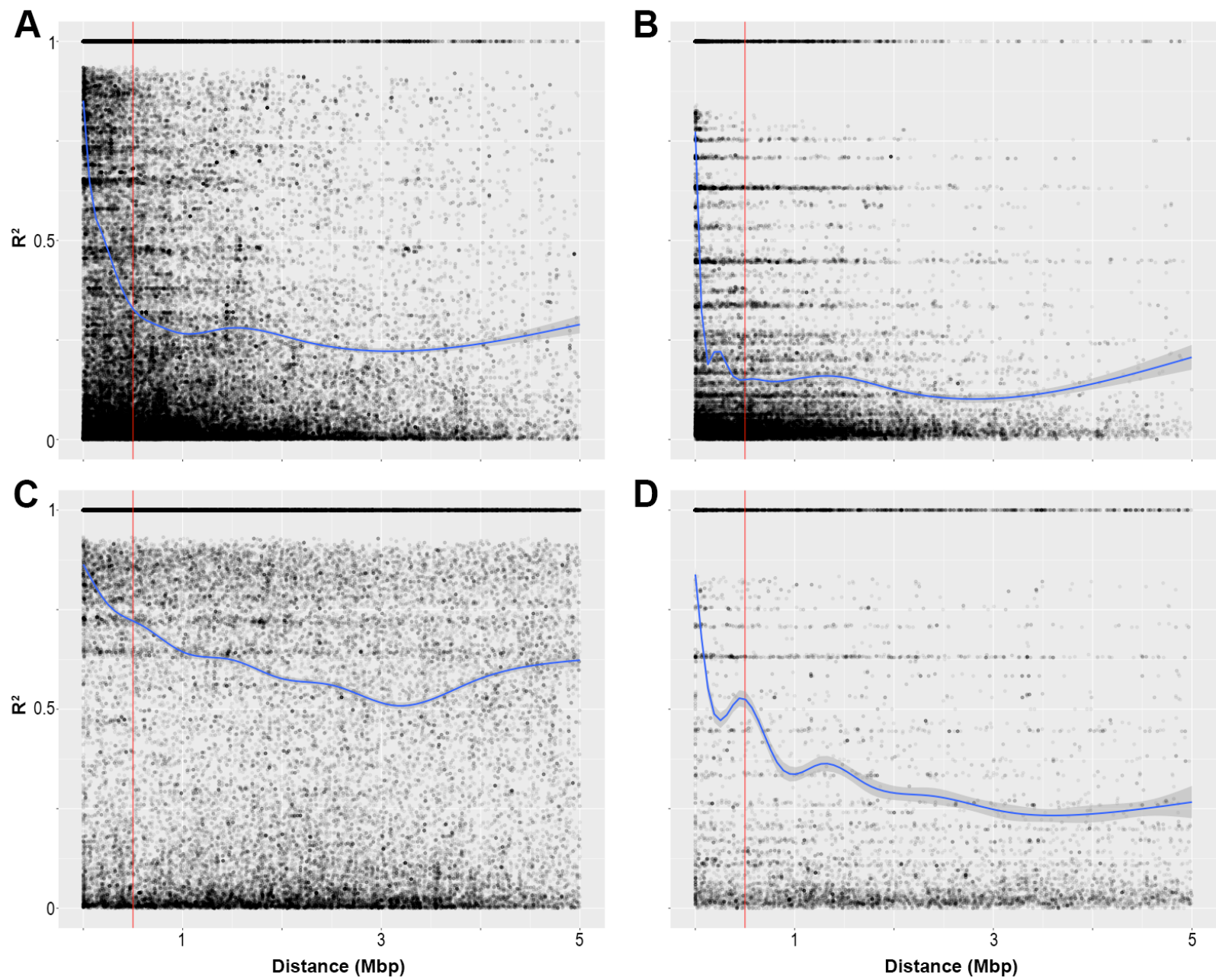

Figure S7. LD-decay plots.

Supplement: Supplementary file 7 [file EVA-12-78-s008.pdf]

**Figure S8. Genome scans for deviation from selectively neutral evolution.**

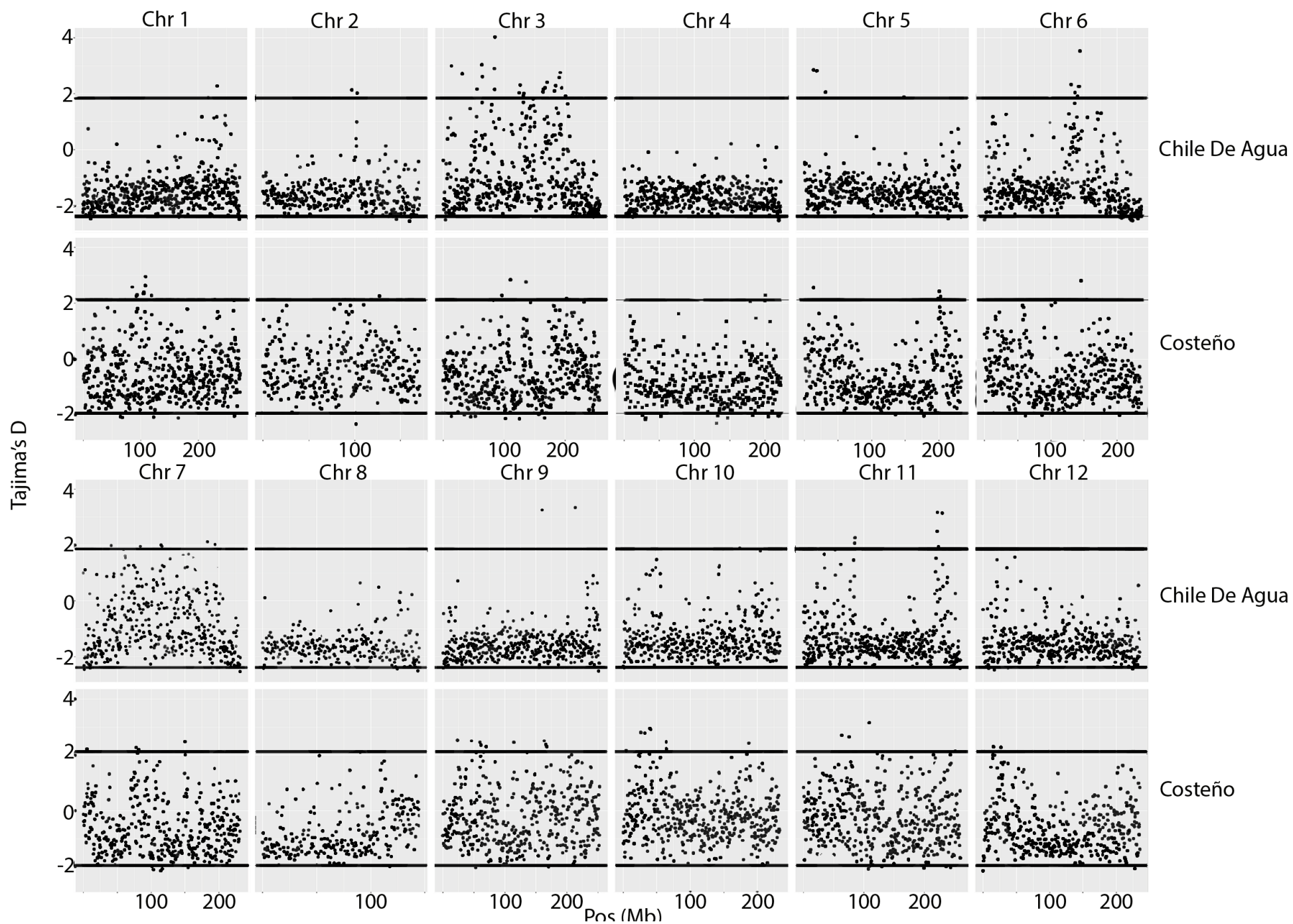

Supplement: Supplementary file 8 [file EVA-12-78-s009.pdf]

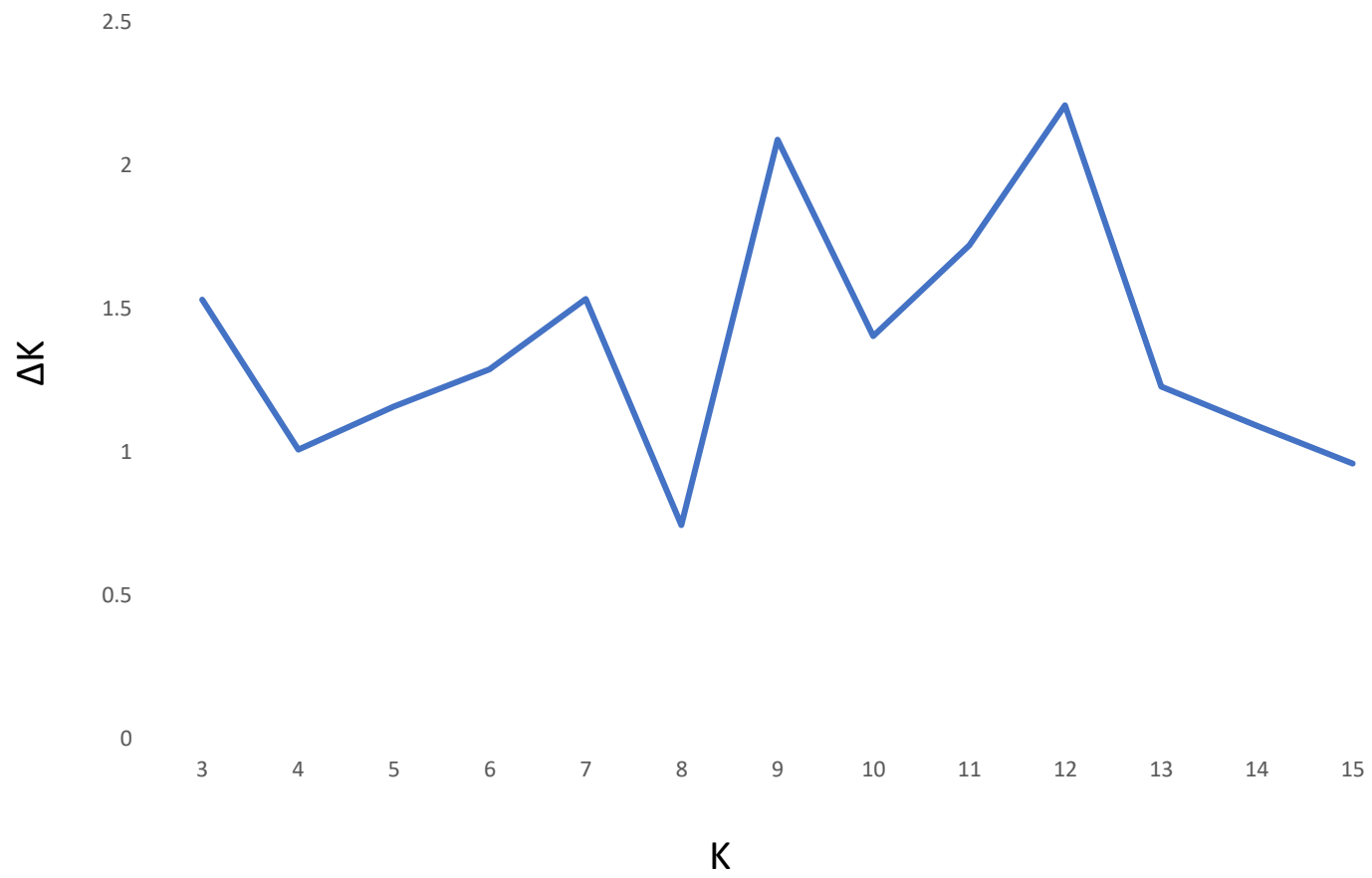

**Figure S9.  $\Delta K$  plot for global collection.**

Supplement: Supplementary file 9 [file EVA-12-78-s010.pdf]
