# Supplementary material for: Genome‐wide genotyping of a novel Mexican Chile Pepper collection illuminates the history of landrace differentiation after Capsicum annuum L. domestication
Source: Evol Appl. 2018 Jul 11;12(1):78–92. doi: 10.1111/eva.12651 (PMC6304684; doi:10.1111/eva.12651)
Supplement: Supplementary file 13 [file EVA-12-78-s002.pdf]

Appendix S1. Detailed summary of SNP-calling steps.

Data Management and Filtration

1.) FastqToTagCount

a. Purpose: to identify and count the number of identifiable tags generated from each lane of the Illumina sequencer. The FastqToTagCount plugin does not parse barcodes and assign them to individual plants. Instead, it merely checks each sequence read for the barcode and cutsite, keeps only those reads where the first 64 nucleotides of the gDNA tag are free of Ns or additional ApeKI cutsites, and truncates the reads - removing everything after those 64 nucleotides - to avoid reading into the Illumina adapter on the other end.

b. Inputs/parameters:

   i. FASTQ read+quality data returned by Illumina sequencer

   ii. Key file relating barcodes to wells on a plate (and thus individual plants)

c. Outputs:

   i. TagCount file, isolating the readable tags returned by each lane of the sequencer, not yet assigned to individual plants

2.) MergeMultipleTagCount

a. Purpose: to merge the same tags when found multiple times, in order to create a reference list of all unique tags in the study.

b. Inputs/parameters:

   i. TagCount file, generated by FastqToTagCount.

    ii. Threshold for the minimum number of times the same tag sequence must be read. Tags not meeting this threshold are removed from the dataset. We set this at one, in order to avoid excluding whole tags with a single base-pair sequencing error and otherwise informative data. Instead of removing whole tags with low depth, we elected to filter out SNPs covered at low depth later in the pipeline.

  c. Output:
    i. Master tag list containing all unique gDNA tags and the number of times they appear in the entire population, which - after conversion to FASTQ - can be referenced in the TagsByTaxa file produced by SeqToTBTHDF5 (4).

3.) TagCountToFastq (Step 1: read data management and filtration)
  a. Purpose: to convert the master tag list from (2) to a format in which they can be aligned by BWA (6) and SeqToTBTHDF5 (4).
  b. Inputs/parameters:
    i. Master tag list
  c. Outputs:
    i. Converted master tag list

Barcode Parsing to Identify Read Source

4.) SeqToTBTHDF5
  a. Purpose: to parse barcodes and relate tags in the Master tag list to the corresponding individual.
  b. Inputs/parameters:

i. Original FASTQ sequences

ii. Converted master tag list returned by MergeMultipleTagCount, reformatted by TagCountToFastq

iii. Barcode key file

iv. Enzyme (ApeKI)

c. Output:

i. TagsByTaxa (TBT) file for each plate stored in the Hierarchical Data Format v5 (.h5). Relates the tags contained in the master tag list to the individual plants whose genomes contain that tag sequence (as determined by the attached barcode).

5.) MergeTagsByTaxa

a. Purpose: merge the TBT files from each plate into a single file for the experiment. Because the same barcodes were used for each of the two plates, it is important that data from the two plates remain separate until this point.

b. Inputs/parameters:

i. TBT files returned by running 1-3 on each plate.

c. Output:

i. TBT file for each plant, including all greenhouse plants for the whole experiment.

SNP-Calling

6.) Burrows-Wheeler Aligner (BWA)

a. Purpose: align all tags to the reference genome in preparation for SNP-calling

b. Inputs/parameters:

i. *C. annuum* cv CM334 reference genome (Kim et al. 2014)

ii. Number of CPU threads to use: specified 4 threads

iii. Master tag list in fastq format

iv. “samse” option to indicate single-end reads

c. Output:

i. SAM alignment file

7.) SAMConverter

a. Purpose: convert the BWA alignment file to TASSEL’s TagsOnPhysicalMap (TOPM) format.

b. Inputs/parameters

i. SAM alignment file returned by BWA

c. Output

i. TagsOnPhysicalMap file for SNP calling

8.) ModifyTagsByTaxa

a. Purpose: “pivot” the TBT file returned by MergeTagsByTaxa so that it is in the proper orientation for use by DiscoverySNPCaller

b. Inputs/parameters

i. TBT file returned by MergeTagsByTaxa

ii. –p parameter to designate pivoting as the modification

c. Output

i. pivoted TBT file

9.) DiscoverySNPCaller

a. Purpose: call SNPs that pass a final filtration step, and mark the SNP locations on the genome. Does not yet assign genotypes to individuals.

b. Inputs/parameters

   i. pivoted TBT file from ModifyTagsByTaxa

   ii. TOPM-formatted, reference-aligned master tags list from SAMConverter

   iii. Mexican *chiltepin* (wild chile pepper) reference genome (Qin et al. 2014)

   iv. –mnF (minimum inbreeding coefficient) parameter set to -0.1. This was because some outcrossing was expected in the population, and thus we avoided removing SNPs where this was low. This option is only set above -0.1 for highly homozygous inbred lines (Glaubitz et al. 2014).

   v. –mnMAF (minimum minor allele frequency) set at 0.01, including SNPs with minor allele frequencies of >1% to exclude sequencing errors.

   vi. –mnMAC (minimum minor allele count) set at three, including SNPs where the minor allele was read at least three times to exclude sequencing errors.

c. Output

   i. TOPM file containing all variants in the population, aligned to the reference genome

10.) ProductionSNPCaller (Step 3: Calling SNPs)

a. Purpose: Assign genotypes to individuals

b. Inputs/parameters:

   i. Original fastq files returned by Illumina sequencer

   ii. TOPM file with aligned variants, returned by DiscoverySNPCaller

   iii. –e (enzyme) parameter specified as ApeKI

c. Output

i. Genotype table for all individuals, in HDF5 format, ready for viewing in the TASSEL GUI, whereupon they can be exported into the desired format. In our case, we selected the Variant Call Format (VCF) for later use with VCFTools (Danecek et al. 2011).
