# Supplementary material for: Genome‐wide genotyping of a novel Mexican Chile Pepper collection illuminates the history of landrace differentiation after Capsicum annuum L. domestication
Source: Evol Appl. 2018 Jul 11;12(1):78–92. doi: 10.1111/eva.12651 (PMC6304684; doi:10.1111/eva.12651)

Figure S6. Close-up map of Central Valleys Chile de Agua collection sites.

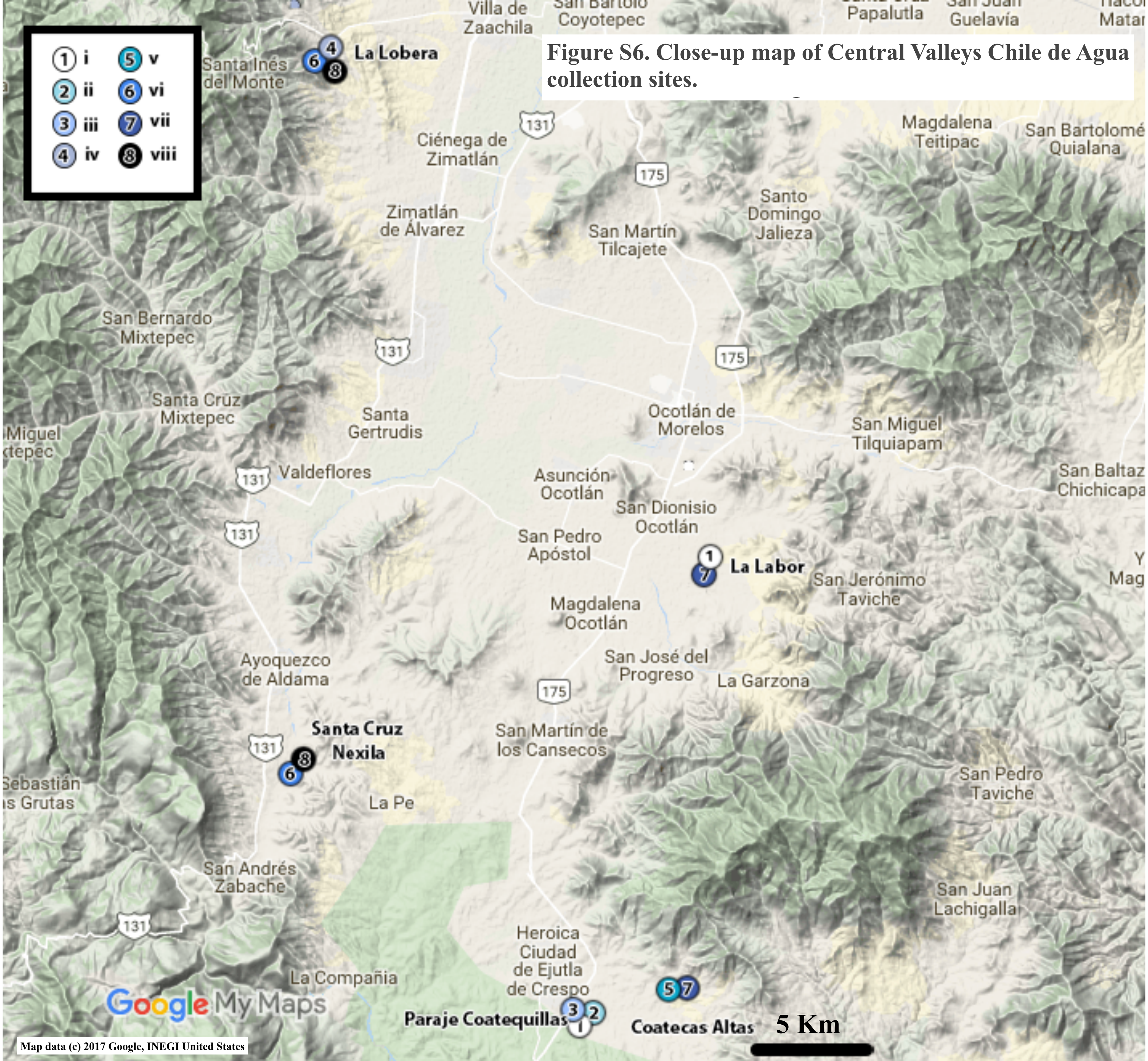

Supplement: Supplementary file 6 [file EVA-12-78-s007.pdf]
